# Screening for tuberculosis infection among newly arrived asylum seekers: Comparison of QuantiFERON^®^TB Gold with tuberculin skin test

**DOI:** 10.1186/1471-2334-8-65

**Published:** 2008-05-14

**Authors:** Brita Askeland Winje, Fredrik Oftung, Gro Ellen Korsvold, Turid Mannsåker, Anette Skistad Jeppesen, Ingunn Harstad, Berit Tafjord Heier, Einar Heldal

**Affiliations:** 1 Division of Infectious Disease Epidemiology, Norwegian Institute of Public Health, 0403 Oslo, Norway; 2 Baerum Municipal Health Department, Tanum asylmottak, 1304 Sandvika, Norway; 3 Faculty of medicine, Norwegian University of Science and Technology, 7941 Trondheim, Norway

## Abstract

**Background:**

QuantiFERON^®^TB Gold (QFT) is a promising blood test for tuberculosis infection but with few data so far from immigrant screening. The aim of this study was to compare results of QFT and tuberculin skin test (TST) among newly arrived asylum seekers in Norway and to assess the role of QFT in routine diagnostic screening for latent tuberculosis infection.

**Methods:**

The 1000 asylum seekers (age ≥ 18 years) enrolled in the study were voluntarily recruited from 2813 consecutive asylum seekers arriving at the national reception centre from September 2005 to June 2006. Participation included a QFT test and a questionnaire in addition to the mandatory TST and chest X-ray.

**Results:**

Among 912 asylum seekers with valid test results, 29% (264) had a positive QFT test whereas 50% (460) tested positive with TST (indurations ≥ 6 mm), indicating a high proportion of latent infection within this group. Among the TST positive participants 50% were QFT negative, whereas 7% of the TST negative participants were QFT positive. There was a significant association between increase in size of TST result and the likelihood of being QFT positive. Agreement between the tests was 71–79% depending on the chosen TST cut-off and it was higher for non-vaccinated individuals.

**Conclusion:**

By using QFT in routine screening, further follow-up could be avoided in 43% of the asylum seekers who would have been referred if based only on a positive TST (≥ 6 mm). The proportion of individuals referred will be the same whether QFT replaces TST or is used as a supplement to confirm a positive TST, but the number tested will vary greatly. All three screening approaches would identify the same proportion (88–89%) of asylum seekers with a positive QFT and/or a TST ≥ 15 mm, but different groups will be missed.

## Background

The incidence of tuberculosis in Norway is generally low (6.3/100 000 population in 2006), but high among immigrants from countries where tuberculosis is endemic [1]. Most cases of tuberculosis are due to imported new strains rather than transmission within Norway [2,3]. WHO have estimated the global prevalence of latent tuberculosis infection in 1997 to be 35% for Africa, 44% for Southeast Asia and 15% for Europe [4]. The enormous pool of persons with latent tuberculosis challenges control of tuberculosis in low endemic countries. National guidelines for prevention and control of tuberculosis therefore recommend targeted tuberculin testing and treatment of latent infection [5]. The recommendations have been challenged by the lack of an accurate diagnostic tool. There are well known limitations of the use of tuberculin skin test (TST). In vitro assays based on cellular production of interferon-gamma (IFN-γ) in response to the *M. tuberculosis *specific antigens ESAT-6 and CFP10 have recently been developed. These protein antigens are present in all species of the *M. tuberculosis*-complex (including *M. bovis*), but absent in all vaccine strains of *M. bovis*-BCG (Bacillus Calmette-Guérin) and most non-tuberculosis mycobacteria, except *M. marinum, M. zulgai*, and *M. kansasii*. These tests can therefore diagnose infection with *M. tuberculosis *with a higher specificity [6,7]. One of the IFN-γ release assays, QuantiFERON^®^TB Gold (QFT), offers logistic advantages and may be suitable for routine screening. The assay has been tested in numerous contact investigations, among patients with tuberculosis disease and exposed health care workers, but data are limited for immigrant screening [7-12]. Among 100 immigrants from high prevalence countries attending an outpatient clinic in Italy, 44% and 15% were positive with TST (≥10 mm) and QFT, respectively [13]. Vast health care resources are spent on screening for tuberculosis. The improved specificity of the IFN-γ release assays is important in this context, both on individual and public health level. The aim of the present study was therefore to compare QFT and TST among newly arrived asylum seekers in Norway and to assess the possible role of QFT in routine diagnostic screening for tuberculosis infection in this target group.

## Methods

Asylum seekers undergo mandatory screening for tuberculosis upon arrival in Norway, with TST and for persons over 15 years also chest X-ray [14]. The coverage of screening of asylum seekers is high, as approximately 95% of all asylum seekers arriving in Norway are housed in one central reception centre, Tanum, the first days after arrival. Legal issues, immediate medical needs and screening for tuberculosis are taken care of before the asylum seekers are transferred to more long term facilities elsewhere in Norway. All asylum seekers, 18 years or older, arriving at Tanum reception centre were invited to participate in the study and enrolment was continued until 1000 were included after informed consent. The study period lasted from September 2005 to June 2006. Consenting participants had a QFT test, a questionnaire regarding demographics, previous BCG-vaccination, previous history or known exposure to tuberculosis, in addition to mandatory TST. Observed presence of a scar was considered evidence of BCG-vaccination, and known contact with an infectious case of tuberculosis was reported as exposure. The blood sample for QFT was drawn at the time the TST was read. Data were available for all asylum seekers only on country of origin and sex. In order to assess the representativity of the study group additional data on age and TST results were collected from the mandatory screening of a consecutive sample of 250 persons who did not agree to participate in the study: 125 persons at study start and 125 persons half way through the study. Normal procedures in accordance with national guidelines were followed, regardless of participation in the study. The Regional Ethics Committee for Medical research recommended the study (S-05122) and the Norwegian Data Inspectorate gave permission (23145).

TST was performed according to the Mantoux method with Purified Protein Derivate (PPD): RT 23, 2 TU from SSI, Copenhagen, Denmark. Four experienced nurses applied and read the test. A test was considered positive if the induration was ≥ 6 mm after 72 hours. Reading was double if the indurations were large, showed adverse reactions or were hard to read.

QuantiFERON^®^TB Gold in-tube-test (Cellestis Ltd, Carnegie, Victoria, Australia) was used. One ml of venous blood was drawn into one tube pre-coated with synthetic peptide antigens and one tube without antigens (negative control sample) and transported the same day to the Norwegian Institute of Public Health for analysis. Samples were incubated, processed and stored in accordance with the manufacturer's instructions before harvested plasma was subjected to Enzyme-Linked Immunosorbent Assay (ELISA) analysis, including IFN-γ standard for quantification. The quality of all laboratory analysis and calculation of the results was controlled by using the accompanying QFT analysis software. A sample was considered positive if exceeding the standard cut-off value at 0.35 IU IFN-γ/ml. All positive results were confirmed by re-analysis of the same plasma sample before reported as positive. If it was not possible to reproduce a positive result, the QFT result was reported as non-conclusive and the participant excluded from the study.

Data were entered into Excel locally and later validated at the Norwegian Institute of Public Health. Kappa-statistics, confidence intervals for single samples (Wilson's method) and unpaired samples (Newcombe's method) were calculated in Confidence Interval Analysis Software (CIA – Trevor N Bryant, 2000) [15]. The other statistical analyses were performed in STATA 9.2 (Statacorp, Texas, 77845, USA). Four different uni- and multivariate unconditional logistic regression analyses were performed in order to identify predictors for positive QFT and TST results. The outcome variables were QFT result and TST result ≥ 6 mm, ≥ 10 mm and ≥ 15 mm. Age (in 4 groups using 10 year periods), sex, origin (Europe, Asia, Africa), BCG-vaccination (scar/no scar), previous history of tuberculosis (yes/no) and known exposure (yes/no) were included as independent variables in a preliminary multivariable regression analysis. The independent categorical variables were expressed as dummy variables. The Spearman correlation coefficient was used to check the correlation between all pairs of independent variables. We subtracted one variable at a time using the likelihood ratio test as elimination criterion (p < 0.05). The same approach was used to test the significance of the two-way interaction terms between the independent variables in the final model. The odds ratios calculated from the estimated coefficients in the final models were used to measure the strength of association.

## Results

### Enrolment and characteristics of the study group

A total of 2963 asylum seekers, 18 years and older, arrived in Norway during the study period and 2813 (95%) of them entered Tanum reception centre. Of the 1000 participants enrolled, 88 (8.8%) were later excluded due to: withdrawal (1), missing TST-results (5), no QFT result due to technical routine error during blood sampling or analyses (47), non-reproducible positive result on the first plasma sample (34) and in one case the first analysis showed too high background value in the negative control sample, while the second analysis was positive. There was not enough plasma for a new analysis and this case (1) was excluded due to lack of confirmation of the positive result. Only the exclusion of the latter 35 cases is attributable to the performance of the QFT assay, and many of these cases had results clustered at or very near the IFN-γ cut-off value (0.35 U/ml). Their TST indurations were in the range of 0–22 mm (median 7 mm). This left 912 participants with valid QFT- and TST results. The study group included significantly more men and fewer persons from Europe compared to all arriving asylum seekers (Table 1). By comparing 250 non-participants with the study group, we found no significant differences in size of the TST, presence of BCG scar or age distribution (data not shown). Median age in the study group was 29 years (range 18–67) and the participants arrived from 60 different countries. Main countries of origin were Iraq (168), Somalia (155), Russia (61), Iran (63), Eritrea (59) and Afghanistan (47), representing 61% of the study group. Russian and Serbian origin dominated among the European participants. Among those with African origin, 342 (92%) came from Sub-Saharan Africa. Scar from previous BCG-vaccination was observed in 658 (72%) participants, more frequently among Europeans (87%) and less frequently among Africans (65%).

**Table 1:** Characteristics of total number of asylum seekers and those participating in the study

	Total arrivals at Tanum			Study group		
	No.	%	95% CI	No.	%	95% CI

Total	2813			912		
Sex						
*Male*	1983	*70.5*	68.8 – 72.2	685	*75.1*	72.2 – 77.8
*Female*	830	*29.5*	27.8 – 31.2	227	*24.9*	22.2 – 27.8
Origin						
*Europe*	473	*16.8*	15.5 – 18.2	115	*12.6*	10.6 – 14.9
Asia*	1304	*46.3*	44.5 – 48.2	418	*45.8*	42.6 – 49.1
*Africa*	1021	*36.3*	34.5 – 38.1	371	*40.7*	37.5 – 44.0
*America*	7	*0.2*	0.1 – 0.5	4	*0.4*	0.2 – 1.1
*Unknown*	8	*0.3*	0.1 – 0.6	4	*0.4*	0.2 – 1.1
*Includes Turkey (no = 15 of study group)						

Characteristics of asylum seekers 18 years or older who arrived at Tanum reception centre during the study period September. 19. 2005 to June 12. 2006.

### TST and QFT results

**Figure 1:**
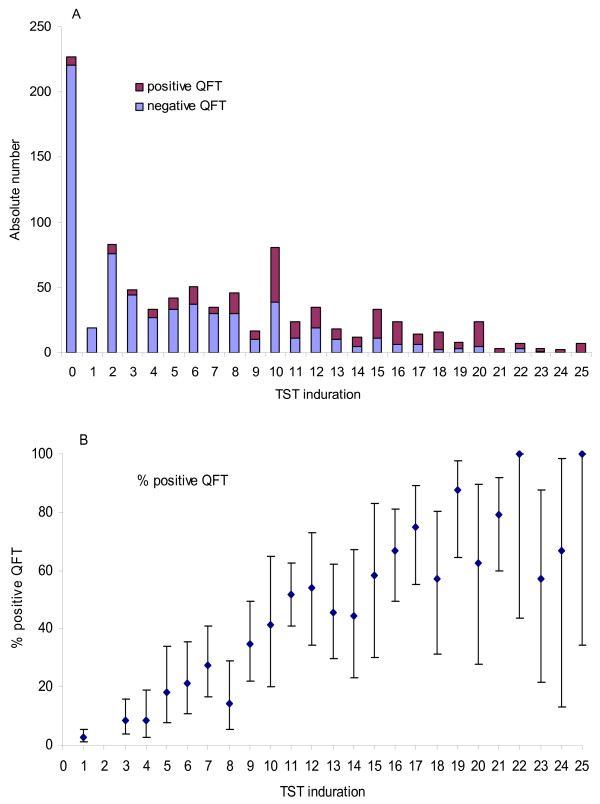
**Distribution of QFT-results by size of TST induration**. Distribution of QFT results by size of TST-induration in absolute numbers (fig 1a) and as percentage of positive QFT tests for each millimetre tuberculin skin test induration with 95% CI (fig 1b), among 912 newly arrived asylum seekers in Norway Sept. 19. 2005 – June12.2006.

A total of 460 (50%) participants tested positive with TST (≥ 6 mm) while 264 (29%) tested positive with QFT. Among the TST positive participants 50% were QFT negative, whereas 7% of the TST negative participants (< 6 mm) tested positive with QFT. Positive TST and QFT tests were most common among African participants (63% and 43%). European participants more often tested positive on TST (52%) than Asian participants (39%), while QFT-results were similar among European and Asian participants, 19% vs 20% respectively. Positive QFT-results were seen with all sizes of TST indurations, but the proportion of positive QFT tests increased with increasing induration size (Figure 1a and 1b). Among the 1000 asylum seekers initially enrolled in the study, five were diagnosed with culture-confirmed tuberculosis. Out of these, two were among participants later excluded from the study; one (TST 15 mm) did not have a QFT-result and one (TST 17 mm) only had a single positive QFT, without a confirmatory test done. Among the remaining three, one patient (TST 9 mm) had a negative QFT result and two were QFT positive (10 and 18 mm TST). All five reported exposure to and symptoms of tuberculosis and had chest x-rays indicative of tuberculosis.

### Agreement between QFT and TST

Overall kappa-values for agreement between QFT and TST were fair to moderate depending on the chosen cut-off values for TST, and they were highest with TST cut-off ≥ 10 mm (Table 2). The agreement (TST ≥ 10 mm) was higher among participants with no scar from BCG: Kappa 0.66 (95% CI 0.56 – 0.77) compared to vaccinated participants: Kappa 0.45 (95% CI 0.37 – 0.52). The agreement was lower for European participants: Kappa 0.32 (95% CI 0.18 – 0.52) than for African: Kappa 0.45 (95%CI 0.36 – 0.54) and Asian: Kappa 0.58 (95% CI 0.48 – 0.67) participants.

**Table 2:** Agreement between TST and QuantiFERON^®^TB Gold for different cut-off values of TST

TST	QFT	TST cut-off ≥ 6 mm	TST cut-off ≥ 10 mm	TST cut-off ≥ 15 mm
		No. (%)	No. (%)	No. (%)

Positive	Positive	232 (25)	190 (21)	104 (11)
Negative	Negative	420 (46)	527 (58)	611 (67)
Positive	Negative	228 (25)	121 (13)	37 (4)
Negative	Positive	32 (4)	74 (8)	160 (18)
Total agreement		652 (72)	717 (79)	715 (78)
Kappa (95% CI)		0.43 (0.37 – 0.49)	0.51 (0.45 – 0.57)	0.39 (0.32 – 0.47)

Agreement between tuberculin skin test and QuantiFERON^®^TB Gold among 912 newly arrived asylum seekers in Norway Sept. 19. 2005 – June 12. 2006 by different cut-off values of tuberculin skin test indurations.

### Predictors of positive tests

Predictors of a positive QFT-result in multivariate regression analysis were age, origin and exposure. With TST ≥ 15 mm as outcome variable gender, age, and origin were predictors (Table 3a and 3b). Adjusting TST cut-off to ≥ 10 mm did not change the predictors, while only age and origin were significant with 6 mm cut-off. Presence of BCG-scar was not a significant predictor for either test, regardless of TST cut-off value. As persons reporting previous history of tuberculosis also reported known exposure these variables were strongly correlated (Spearman's rho 0.657, p < 0.001) and did not fit into the same model.

**Table 3:** Predictors of (a) positive QuantiFERON^®^TB Gold results (b) tuberculin skin test reaction >15 mm

A. Predictors of positive QFT				Univariate		Multivariate	
Variable	Characteristic	Total No.	No. QFT+	OR (95%CI)	p-value	*a *OR (95%CI)	p-value

BCG – scar	no scar	246	68	1	0.53		
	scar	658	196	1.1 (0.8–1.5)			
Sex	male	685	202	1	0.53		
	female	227	62	0.9 (0.6–1.3)			
Age group	18–29 years	471	129	1	< 0.001	1	< 0.001
	30–39 years	291	75	0.9 (0.7–1.3)		1.1 (0.8–1.5)	
	40–49 years	115	40	1.4 (0.9–2.2)		1.8 (1.1–2.9)	
	50+ years	35	20	3.5 (1.8–7.1)		4.6 (2.2–9.5)	
Origin	Asia	418	82	1	< 0.001	1	< 0.001
	Europe	115	22	1.0 (0.6–1.6)		1.0 (0.6–1.7)	
	Africa	371	159	3.1 (2.2–4.2)		3.3 (2.4–4.6)	
Exposure	no	898	255	1	0.01	1	0.02
	yes	14	9	4.5 (1.5–13.7)		4.0 (1.3 – 12.9)	
Previous diagnosis of tuberculosis	no	904	259	1	0.05		
	yes	8	5	4.2 (1 – 17.5)			

*Log likelihood = -502.86642, p-value final model = 0.000, No. of observations in final model = 904*							

B. Predictors of TST ≥ 15 mm				Univariate		Multivariate	

Variable	Characteristic	Total No.	No. TST ≥15 mm	OR (95%CI)	p-value	*a *OR (95%CI)	p-value

BCG – scar	no scar	246	39	1	0.85		
	scar	658	101	1.0 (0.6–1.4)			
Sex	male	685	85	1	< 0.001	1	< 0.001
	female	227	56	2.3 (1.6–3.4)		1.9 (1.3–2.8)	
Age group	18–29 years	471	63	1	0.31	1	< 0.002
	30–39 years	291	44	1.2 (0.8–1.7)		1.5 (1–2.3)	
	40–49 years	115	23	1.6 (1–2.7)		1.9 (1.1–3.3)	
	50+ years	35	11	3.0 (1.4–6.4)		4.3 (1.9–9.5)	
Origin	Asia	418	32	1	< 0.001	1	< 0.001
	Europe	115	21	2.7 (1.5–4.9)		2.4 (1.3–4.5)	
	Africa	371	88	3.8 (2.4–5.8)		3.8 (2.4–6)	
Exposure	no	898	136	1	0.05		
	yes	14	5	3.1 (1–9.4)			
Previous diagnosis of tuberculosis	no	904	138	1	0.10		
	yes	8	3	3.3 (0.8–14.1)			

*Log likelihood = -359.05381, p-value final model = 0.000. No. of observations in final model = 904*							

Results from uni- and multivariate regression analyses with positive QuantiFERON^®^TB Gold (table 3a) and tuberculin skin test reaction >15 mm (table 3b) as outcome variables among 912 newly arrived asylum seekers to Norway, Sept. 19. 2005 – June 12. 2006. Blanks in multivariate analyses means that the variable was not a significant predictor of a positive test. Participants with American or unknown origin were excluded from statistical analysis, due to low number of cases.

### Implications for screening

If QFT is implemented in routine screening the number in need of referral could be reduced by 43% compared with referral based only on a positive TST ≥ 6 mm (50% TST positives compared to 29% QFT positives) (Table 4). The proportion referred is less affected by whether QFT is implemented as a replacement of or as a supplement to TST. Different approaches would identify the same percentage (88–89%) with a positive QFT and/or a TST ≥ 15 mm, but the number of tests required will vary. Changing the TST cut-off value to 10 or 15 mm in a two-step approach will reduce the number tested with QFT at the cost of number identified with latent infection.

**Table 4:** Different approaches to routine screening with tuberculin skin test and QuantiFERON^®^TB Gold

Screening programme approach	Referral criteria	Proportion (%) referred for treatment	Proportion (%) LTBI* cases detected (detection rate)	Proportion (%) non-infected referred for treatment (false positive rate)
Only TST	TST ≥ 6 mm	50	89	42
	TST ≥ 10 mm	34	75	27
	TST ≥ 15 mm	15	47	0
Only QFT	QFT positive	29	88	0
Two step (first TST, then QFT)	TST ≥ 6 mm and QFT positive, or TST ≥15 mm	29	89	0
	TST ≥ 10 mm and QFT positive or TST ≥ 15 mm	25	75	0
	TST ≥ 15 mm (with or without QFT positives afterwards)	15	47	0

*Latent infection is defined as either having a positive QuantiFERON(R)TB Gold test (264 participants) or a tuberculin skin test ≥15 mm and a negative QuantiFERON(R)TB Gold test (37 participants). Effect on screening based on identification of 301 cases of latent tuberculosis infection among 912 newly arrived asylum seekers in Norway Sept. 19. 2005 – June 12.2006

## Discussion

The performance of new and specific blood tests for detecting latent infection has now been studied in several contexts, including contact tracing and screening of defined risk groups like immigrants and immunocompromised persons [7-12]. We have in this work recruited a large number of asylum seekers to compare the performance of TST and QFT and used the results to evaluate the role of QFT in screening immigrants for latent infection when arriving in a low-incidence country. Although only 36% of the eligible asylum seekers agreed to participate, they seemed to be fairly representative of all asylum seekers arriving, even though their countries of origin are changing over time. Fewer female asylum seekers participated, probably because of more stigma and poorer language skills. The 35 cases without a positive confirmatory test were excluded. Including them would potentially overestimate or underestimate the number of QFT positives respectively as their results were uncertain. They represented only approximately 3% of the total number of tests, and in a normal screening setting a new sample would have been collected for re-analysis, which was not feasible in our study. We have assessed the effect of excluding the cases and found no main impact on predictors of positive QFT or on the different screening approaches. The prevalence of latent infection among the asylum seekers was high, both defined by TST (50%) and QFT (29%), as expected in a population predominantly arriving from high prevalence countries. Our findings are similar with WHO estimates based partly on TST studies [4]. Most asylum seekers from Asia came from relatively low prevalence countries, while many Africans came from Somalia where the prevalence is probably very high. Studies involving IFN-γ release assays in high-prevalence settings are few and results vary. One study suggests 80% prevalence of latent infection in urban India, while another reports 56% prevalence among healthy adults in South Africa [16,17].

### Agreement between QFT and TST

Consistent with other studies, we found a moderate agreement between the results of QFT and TST, although differences in design and populations make comparison between such studies difficult [7,9,11,18,19]. Importantly, we observed that the proportion of individuals with positive QFT increased with increasing TST induration, just like the expected proportion of tuberculin reactions actually caused by *M. tuberculosis *would do [20]. A TST reaction of ≥15 mm has been interpreted as high likelihood of true tuberculosis infection [21,22]. Discordant results in our study were found in the range of 21 – 29% depending on the TST cut-off value, but mainly as positive TST and negative QFT in the range of 6–9 mm (72%) and 10–14 mm (49%). The poor specificity of TST in these intervals is a major challenge as most positive TST results (69%) occurred within this range as well as 48% of the positive QFT-results. False positive TST indurations caused by BCG or non-tuberculosis mycobacteria are expected to be moderate and rarely exceeding 15 mm [10,21-23]. Boosted TST reactions or differences in the nature of the immune response measured by the two tests might also explain discordant results [8,24]. In this study the blood sample was drawn at the time when the TST was read. A potential influence of PPD exposure on a QFT test has been investigated, and having a QFT test after TST is advised to be a reliable approach [25]. This is also in agreement with the European Consensus Report, where it is advised that IGRA should be implemented as a supplement following a positive TST [26].

### Possible explanations for discordant results

Previous BCG vaccination; a meta analysis reported increased likelihood of a positive TST in BCG-vaccinated persons within the first 15 years after vaccination [27]. We found no significant effect of previous BCG-vaccination on QFT nor on TST-results in multivariate regression analyses, regardless of TST cut-off 15, 10 or 6 mm. The limited effect of BCG observed in our study might be explained by the fact that vaccination is most often offered to newborns and all our participants were older than 18 years. In addition, scars might be underreported either by being missed or being misinterpreted by the observer, or vaccinated persons might not have developed a scar. Non-vaccinated persons in this study were to a large extent from Somalia, a population where the prevalence of latent infection is high. This might explain the better agreement between the tests for non-vaccinated compared to vaccinated participants. As revaccination policies have been common in some European countries (including Russia) the stronger effect of BCG vaccination might explain the lower agreement found among European participants.

Difference in immunological responses measured by the two tests; tuberculin induces a delayed-type hypersensitivity reaction reflecting a memory response, while the blood tests detect cellular INF-γ release reflecting an effector response to an ongoing infection. Due to these differences in immunological responses it is suggested that IFN-γ release assays detect more than TST those with recent and persistent infection [8,18]. Differences in immunological responses can partly explain discordance and might have important clinical and programmatic implications, as recent infection is more likely to progress to active disease and preventive treatment therefore indicated. A recent publication indicates at least equivalent sensitivity of QFT compared to TST for predicting progression to tuberculosis [28]. The lack of an age associated increase in discrepant results between QFT and TST (cut-off 15 mm) in Table 3 could indicate that many of the participants have recent infection. There is also some ongoing research to determine whether the level of IFN-γ response to the specific *M. tuberculosis *antigens may be meaningful for predicting the outcome of latent infection [29].

Sensitivity related issues; among participants with a strongly positive TST (≥ 15 mm) we found that 26% (37 out of 141) had a negative QFT test. High proportions of discrepant results in this group has also been observed in other studies [17,30,31]. Among immunocompetent persons the sensitivity of tuberculin is regarded as very high, while sensitivity of QFT for detection of latent infection has been variable (75 – 97%) depending on the study population and design [18,32,33]. Among patients with tuberculous disease confirmed by culture, sensitivity measurements for QFT varies (70–90%) [7,10,34,35]. A recent study reported 13% indeterminate and 20% negative QFT results among patients with culture confirmed tuberculosis [34]. The fact that we found one QFT negative individual among four participants with confirmed active disease demonstrates the need for careful evaluation of test results. The main purpose of TST and QFT used in screening is to identify persons with latent infection eligible for preventive treatment. Screening of symptoms and chest x-ray would have identified all five asylum seekers with active tuberculosis in our study. Among participants with negative TST, we found that 7% (32 out of 452) had a positive QFT. This might reflect false negative TST results as the IFN-γ release assays have shown to have higher sensitivity than TST among immunocompromised individuals [34,36]. The risk of future progression to active disease in TST negative and QFT positive adults should be studied in large, prospective studies. Due to the lack of a positive control for a functional immune response (mitogen stimulation) in the QFT assay available when the study was conducted, the general immune status of the participants were not known. This is a limitation of the study and may in cases of immuno-suppression have lead to false negative results with respect to both QFT and TST. However, recent information on the performance of the QFT test in immuno-compromised individuals demonstrates that only severe immune-suppression with low CD4 counts may lead to indeterminate results and potential false negative results in case of no positive mitogen control [37-40]. Based on surveillance of hiv-infection in Norway we have no reasons to believe that severe immuno-suppression was affecting more than very few cases, if any at all, among our participants. On this basis, we do not think that the lack of a positive mitogen control in this study represents any major problem for interpretation of the results. Exposure to *M. bovis *might result in a QFT test that is false positive with respect to *M. tuberculosis*. As clinical management of cases with tuberculous disease is the same for all species included in the *M. tuberculosis*-complex, this does not affect the screening approach.

### Predictors of positive tests

The multivariate analysis showed similar significant risk factors for positive QFT and TST >15 mm. Country of origin and increase in age were both significantly associated with both positive QFT and TST ≥15 mm, reflecting accumulated exposure time and background prevalence in country of origin. Exposure to an infectious case of tuberculosis was significantly related to QFT, but not to TST, as found in other studies [7-9,41-44]. Previous history of tuberculosis was not a significant predictor for either test as it correlated with exposure.

### Implications for screening in Norway

There is no gold standard test for latent infection. Both positive QFT results and TST>15 mm (independent of each other) have been suggested to accurately identify latent infection and also predict progression to active disease [21,23,28] We have, for the purpose of assessing the use of QFT in screening, defined latent tuberculosis infection as having a positive QFT test regardless of TST induration or a TST ≥ 15 mm and a negative QFT. In this study 301 participants are defined as having latent tuberculosis infection; the 264 QFT positive participants and the 37 with a TST ≥ 15 mm and a negative QFT. Based on this definition, a more specific test than TST for diagnosis of latent infection is clearly needed as TST with cut-off at 6 mm or 10 mm results in referral of 42% or 27% false positive cases, respectively, while a cut-off of 15 mm will detect less than half of the actual cases. As it is neither feasible nor cost effective to screen all asylum seekers with both tests, the options are to replace TST with QFT (one step approach) or to use QFT to confirm a positive TST (two step approach). A one step approach with only QFT has logistic advantages and referral based on a false positive TST is avoided. The strength of this approach would be the inclusion of the TST negative among the QFT positive participants (7%), a presumable high-risk group for active disease and cases lost in a two step approach. The two step approach allows for referral procedures based on both tests and it is recommended in a European consensus report from 2006 [26]. This strategy follows normal procedures for screening with a first test with high sensitivity followed by a second test with high specificity. The two-step approach identifies and refers all TST positive cases with a positive QFT-result and also the 12% proportion with TST ≥ 15 mm and a negative QFT, which would be lost in a one step approach (Table 4). The approach of using TST ≥ 6 mm as cut-off value, would identify a similar proportion (88–89%) of persons defined as having latent infection even though it would not be the same persons referred. By implementing QFT the number referred for preventive treatment would be reduced by 43%, independent of whether the one- or two step approach is chosen. Adjusting TST cut-off value to 10 or 15 mm would reduce the number referred to 25% and 15%, respectively, but lost cases with latent infection would consequently increase to 25% and 53%. Whether a one step approach with only QFT is feasible depends also on laboratory capacity and the cost implications of the different screening approaches need further study. Based on the present study the Norwegian Institute of Public Health and its Tuberculosis Advisory Committee have recommended to the Ministry of Health the use of QFT in a two step approach for screening of immigrants.

## Conclusion

The prevalence of latent infection among asylum seekers was high, both defined by TST (50%) and QFT (29%), and with the expected discordance between the two test methods. The increased specificity of QFT is a very important improvement in a screening context as the number of persons referred for preventive treatment would be reduced by 43% independent of whether the one- or two-step approach is implemented, compared to referral based on TST (≥ 6 mm) alone. All three screening approaches would identify a similar proportion (88 – 89%) of those defined as having latent infection, even though it would not be the same persons referred. As only 0.5% of the study group had active tuberculosis disease on arrival, identification of persons with latent infection for referral and assessment for preventive treatment remains a key objective of the screening on entry. Identification of active disease also relies on screening of symptoms and chest x-ray.

## Competing interests

The authors declare that they have no competing interests.

## Authors' contributions

BAW has been responsible for study design, planning, data collection, data analysis and interpretation of data and the writing process, FO has been responsible for the laboratory components of the study and has made substantial contributions to the writing process, GEK carried out laboratory work and contributed to interpretation of data, TM contributed to planning of the study, interpretation of data and input in the writing process, ASJ has contributed to study design, data collection and quality assessment of data, IH participated in data collection and analyses of results, BH contributed to statistical analyses of data and EH contributed to planning of the study, data interpretation and analyses of results as well as giving substantial input in the writing process. All authors read and approved the final manuscript.

## Pre-publication history

The pre-publication history for this paper can be accessed here:


